# Whole-genome analysis of piscine reovirus (PRV) shows PRV represents a new genus in family *Reoviridae* and its genome segment S1 sequences group it into two separate sub-genotypes

**DOI:** 10.1186/1743-422X-10-230

**Published:** 2013-07-11

**Authors:** Molly JT Kibenge, Tokinori Iwamoto, Yingwei Wang, Alexandra Morton, Marcos G Godoy, Frederick SB Kibenge

**Affiliations:** 1 Department of Pathology and Microbiology, Atlantic Veterinary College, University of Prince Edward Island, 550 University Ave., Charlottetown, PEI C1A 4P3, Canada; 2 Department of Computer Science and Information Technology, University of Prince Edward Island, 550 University Ave., Charlottetown, PEI C1A 4P3, Canada; 3 Raincoast Research Society, Box 399, 390 1st Street, Sointula, BC V0N 3E0, Canada; 4 Centro de Investigaciones Biológicas Aplicadas (CIBA), Diego de Almagro Norte 1013, No. 10, Puerto Montt, Chile; 5 Universidad San Sebastián. Facultad de Ciencias, Lago Panguipulli 1390, Puerto Montt, Chile; 6 ETECMA, Diego de Almagro Norte 1013 No. 10, Sector Cardonal, Puerto Montt, X Región, Chile

## Abstract

**Background:**

Piscine reovirus (PRV) is a newly discovered fish reovirus of anadromous and marine fish ubiquitous among fish in Norwegian salmon farms, and likely the causative agent of heart and skeletal muscle inflammation (HSMI). HSMI is an increasingly economically significant disease in Atlantic salmon (*Salmo salar*) farms. The nucleotide sequence data available for PRV are limited, and there is no genetic information on this virus outside of Norway and none from wild fish.

**Methods:**

RT-PCR amplification and sequencing were used to obtain the complete viral genome of PRV (10 segments) from western Canada and Chile. The genetic diversity among the PRV strains and their relationship to Norwegian PRV isolates were determined by phylogenetic analyses and sequence identity comparisons.

**Results:**

PRV is distantly related to members of the genera *Orthoreovirus* and *Aquareovirus* and an unambiguous new genus within the family *Reoviridae*. The Canadian and Norwegian PRV strains are most divergent in the segment S1 and S4 encoded proteins. Phylogenetic analysis of PRV S1 sequences, for which the largest number of complete sequences from different “isolates” is available, grouped Norwegian PRV strains into a single genotype, Genotype I, with sub-genotypes, Ia and Ib. The Canadian PRV strains matched sub-genotype Ia and Chilean PRV strains matched sub-genotype Ib.

**Conclusions:**

PRV should be considered as a member of a new genus within the family *Reoviridae* with two major Norwegian sub-genotypes. The Canadian PRV diverged from Norwegian sub-genotype Ia around 2007 ± 1, whereas the Chilean PRV diverged from Norwegian sub-genotype Ib around 2008 ± 1.

## Background

The newly discovered piscine reovirus (PRV) belongs to the family *Reoviridae*, subfamily *Spinareovirinae*[1], probably in a new reovirus genus that is equally distant to the genera *Orthoreovirus* and *Aquareovirus*[2], although with 10 genome segments, PRV is like members of the genus *Orthoreovirus* and unlike the genus *Aquareovirus* with 11 segments. The *Orthoreovirus* genus can be divided into the fusogenic and non-fusogenic orthoreoviruses based on the ability of the fusogenic orthoreoviruses to induce cell-cell fusion during infection resulting in syncytium formation [3] by virtue of possession of a fusion-associated small transmembrane (FAST) protein [4]. Whereas the non-fusogenic orthoreoviruses, *Mammalian Orthoreovirus* (MRV), are not clinically significant [5], the fusogenic orthoreoviruses *Nelson Bay virus* (NBV) [6] and *Baboon Orthoreovirus* (BRV) [7] that infect primates, *Avian Orthoreovirus* (ARV) [8] that infect birds, and *Reptilian Orthoreovirus* (RRV) [9] that infect reptiles, have been shown to cause significant and often fatal disease. Most recently, PRV has been shown to be more closely related with recognized orthoreoviruses than with recognized aquareoviruses, and does not encode a FAST protein and is therefore non-fusogenic [10].

PRV is associated with heart and skeletal muscle inflammation (HSMI) [2]; an emerging disease of marine-farmed Atlantic salmon [11], first recognized in 1999 in western Norway [12] and subsequently in Scotland [13]. PRV has also been detected by real-time reverse transcription quantitative polymerase chain reaction (RT-qPCR) at a low prevalence in wild Atlantic salmon “*S. salar*” [2] and in certain marine fish species (Atlantic herring *“Clupea harengus”,* Capelin “*Mallotus villosus*”, Atlantic horse mackerel “*Trachurus trachurus*”, and Great silver smelt “*Argentina silus”*) along the coast of Norway [14]. PRV was also detected in 3% of anadromous trout (sea-trout) “*Salmo trutta*” tested, but not in anadromous Arctic char “*Salvelinus alpinus*” [15]. PRV is ubiquitous in Norwegian salmon farms [16,17], but there is a significant increase in the viral load and tissue distribution during outbreaks of HSMI [2,18]. The virus can be propagated in the GF-1 cell line [19], derived from the tissue of orange-spotted grouper, *Epinephelus coioides*[20], and cardiac and skeletal muscle pathology typical of HSMI can be reproduced in naïve Atlantic salmon by experimental inoculation with the supernatant from cell culture passaged PRV [19]. Most recently, it has been reported that serum enzymes creatine kinase and lactate dehydrogenase, associated with cardiac injury in humans [21], are significantly correlated with HSMI histopathology in Atlantic salmon [22]. Other reports doubt the pathogenicity of PRV, describing PRV as an opportunistic virus [23,24] or non-pathogenic virus [15]. The virus has been detected in marine-farmed Atlantic salmon in Chile [25,26]. There is anecdotal evidence that it is also present in farmed Atlantic salmon and wild Pacific salmon in British Columbia-Canada [27], where 75% of 300 farm salmon reportedly tested positive for PRV [27] but no sequence information was reported.

The PRV genome comprises at least 10 dsRNA segments including three large (L), three medium (M), and four small (S) size-class RNA genome segments [2]. To date only two “isolates”, both from marine-farmed Atlantic salmon from Norway, have been sequenced on all 10 genomic segments by high-throughput pyrosequencing of clinical samples: Reovirus sp. Salmo/GP-2010/NOR from HSMI [2], and CMS PRV from a CMS outbreak [24]. However, only the Salmo/GP-2010/NOR sequences are accessible from the GenBank Database (GenBank accession numbers GU994013-GU994022). The coding assignments of genomic segments S1 and S4 initially reported to encode proteins with no identified homologs in orthoreoviruses and aquareoviruses [2], were recently shown to be reversed such that S1 encodes the major outer capsid protein, Outer clamp protein (σ3 and VP7 in MRV and aquareoviruses, respectively), and S4 encodes the virus attachment protein, Outer fiber protein (σ1 in MRV, which is absent in aquareoviruses) [10]. A further complication is that several sequences of Norwegian PRV isolates were deposited in the GenBank database as S4 sequences [18,28] but correspond to S1 sequences [2, this study], and sequences of the remaining 9 genomic segments for these virus isolates have not been reported.

The sequence data available for PRV strains are limited, with no genetic information on this virus outside of Norway, and none from wild fish despite the economical impact of HSMI on salmon aquaculture and the potential for transmission of PRV to wild salmon populations or from wild salmon to farmed salmon.

The primary goal of the present study was to determine the genetic diversity among PRV strains detected in tissue samples obtained from fish in western Canada, and in Chile, and their relationship to known Norwegian PRV sequences. We also attempted to sequence the complete genomes of three “isolates”, two Canadian and one Chilean to obtain more information about the taxonomic assignment of PRV.

## Results and discussion

### Amplification and sequencing of cDNA of genomic segments of PRV from fish samples

Piscine reovirus was readily detected by RT-qPCR during testing at the Atlantic Veterinary College laboratory in fish tissue samples from western Canada, and at the ETECMA diagnostic laboratory in fish tissue samples from Chile (data not shown). Consistent with observations elsewhere [2,18], PRV is ubiquitous in marine-farmed salmon. PRV was consistently detected in gill tissue, identifying the gills as suitable target tissue and likely a primary transmission route for PRV. This is consistent with *Orthoreovirus*, which are commonly isolated from enteric and respiratory tract tissues [1].

Ten samples from western Canada with either low Ct values or unique case histories, host species, and sampling times listed in (Additional file 1: Table S1a) were selected for amplification and cloning of cDNA of viral genome segments. Four additional samples for which the 3′ portion of genome segment L1 had been PCR-amplified during the original testing for PRV (Table 1), were included in the analysis of PRV sequences.

**Table 1:** List of new piscine reovirus (PRV) “isolates” from Canada and Chile

**PRV “isolate”**	**Fish species**	**Source**
23	Atlantic salmon	Farmed, Canada
163	Atlantic salmon	Farmed, Canada
167	Atlantic salmon	Farmed, Canada
177	Atlantic salmon	Farmed, Canada
185	Atlantic salmon	Farmed, Canada
196	Atlantic salmon	Farmed, Canada
209	Atlantic salmon	Farmed, Canada
321	Atlantic salmon	Farmed, Canada
333	Cutthroat trout	Wild, Canada
340	Cutthroat trout	Wild, Canada
358	Atlantic salmon	Farmed, Canada
371	Atlantic salmon	Farmed, Canada
468	Chum salmon	Wild, Canada
480	Steelhead trout	Farmed, Canada
CGA337	Atlantic salmon	Farmed, Chile
CGA558	Atlantic salmon	Farmed, Chile
CGA8857	Atlantic salmon	Farmed, Chile
CGA280-5	Atlantic salmon	Farmed, Chile

**Figure 1:**
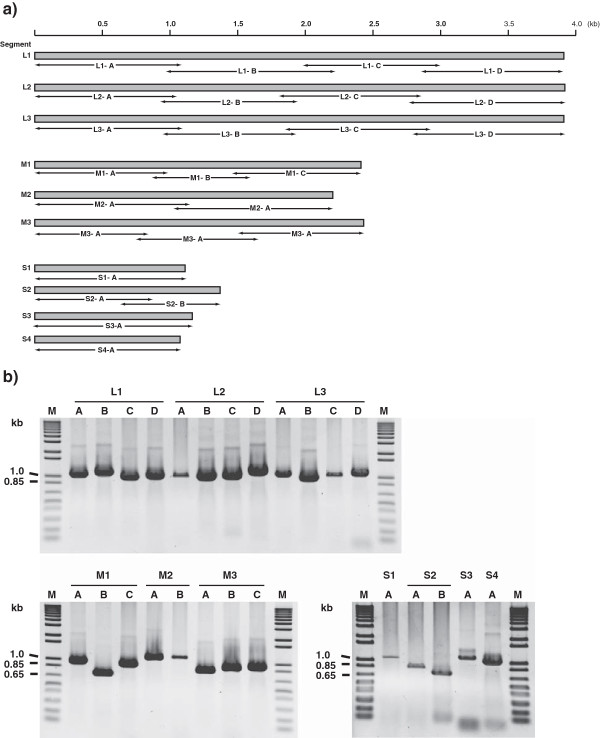
**RT-PCR amplification and sequencing strategy. (a)** Schematic illustration of viral genome segments of piscine reovirus (PRV) RT-PCR targets for nucleotide sequencing. **(b)** Gel electrophoresis of amplified products obtained from the RT-PCR for Canadian PRV isolate 358. The PCR primers are listed in Additional file 5: Table S4. Lane M denotes Molecular weight marker.

Figure 1 shows the RT-PCR amplification and sequencing strategy used for the PRV genome segments, based on Canadian “isolate” 358. The new PRV nucleotide sequences (Additional file 2: Table S2) are available through the GenBank database [29]. The complete PRV genome (10 segments) was amplified from 2 of 10 samples. Additional partial or full-length sequences were also obtained on PRV genomic segments L1 (12 samples), L2 (1 sample), L3 (2 samples), M1 (4 samples), M2 (2 samples), M3 (4 samples), S1 (4 samples), S2 (3 samples), S3 (1 sample), and S4 (1 sample). PRV sequences were obtained from four different western Canada fish species (Atlantic salmon “*Salmo salar*”, Cutthroat trout “*Oncorhynchus clarkii*”, Steelhead trout “*Oncorhynchus mykiss*”, and Chum salmon “*Oncorhynchus keta*”) (Table 1 and Additional file 2: Table S2). Failure to amplify transcripts from all PRV positive samples was attributed to variation in viral loads. It has been reported that fish are capable of reducing the viral load by the end of the production cycle [18]. The differences in RT-PCR amplification could be due to differences between the PRV “isolates”. It is also possible that variations in transcription levels of different virus genes, and efficiency of PCR of the different targets contributed to the inability to amplify all 10 genome segment transcripts in some of the samples.

Among the Chilean PRV positive samples, 6 fish individually sampled from the same farm with low Ct values were selected for amplification of all 10 viral genome segments; sequences from one of these samples was used in the analysis. These were all fish kidney samples, which had significantly lower Ct values (Additional file 1: Table S1b) compared to the fish gill samples from Canada (Additional file 1: Table S1a). Three additional sequences on PRV genomic segments L3 (3 samples) and S1 (2 samples) were already available and were included in the analysis, for a total of 4 Chilean PRV “isolates” (Table 1 and Additional file 2: Table S2).

(Additional file 2: Table S2) shows the nucleotide and amino acid sequence identities of the new PRV isolates when compared to the single Norwegian PRV isolate, Salmo/GP-2010/NOR (GenBank accession numbers GU994013-GU994022). The largest nucleotide sequence differences between Canadian and Norwegian PRV strains are on segments M2 and S1 (96-97% sequence identity). However, at the amino acid sequence level, our analysis shows that Canadian and Norwegian PRV strains are most divergent in the S1 encoded proteins, the major outer capsid protein (Outer clamp protein) and the non-structural protein p13, and the S4 encoded virus attachment protein (Outer fiber) [10]. The difference on the S4 protein is very interesting as it consists of a variable region of 18 residues at the C-terminus. This work is the first report of genomic analysis of PRV strains detected in tissue samples obtained from fish outside of Norway, extending the current geographical range of the characterized virus to both North and South America.

### The PRV conserved terminal nucleotide sequences

Conserved terminal nucleotide sequences are useful for reovirus classification [1]. Palacios *et al.*[2] reported the complete genome sequence of the Norwegian PRV isolate Salmo/GP-2010/NOR including the conserved nucleotides at the 5′ end and the 3′ end of the genome (5′-GAUAAA/U------UCAUC-3). Table 2 compares these conserved terminal sequences to those of members of the *Orthoreovirus* and *Aquareovirus* genera. The conserved nucleotides 5′-GAUAAA/U were present at the 5′ ends in all the positive strands of each of the 10 genome segments of PRV, and are unique to PRV, whereas the 3′ conserved termini UCAUC-3′ are also conserved between PRV, and the *Orthoreovirus* and *Aquareovirus* genera (Table 2).

**Table 2:** **Conserved terminal nucleotide sequences (positive strand) of PRV,*Orthoreovirus*, and*Aquareovirus*genera genome segments**

**Reovirus genus**	**Reovirus species/strain**	**Conserved terminal nucleotide sequences**	
		**5′ terminal nucleotides**	**3′ terminal nucleotides**
PRV	Salmo/GP-2010/NOR [2]*	5′-GAUAAA/U	UCAUC-3′
Orthoreovirus genus	*Avian orthoreovirus*-138 [1]*	5′-GCUUUUU	UCAUC-3′
	*Nelson Bay orthoreovirus*[1]*	5′-GCUUUA	UCAUC-3′
	*Mammalian orthoreovirus* -1La [1]*	5′-GCUA	UCAUC-3′
	*Baboon orthoreovirus*[1]*	5′-GUAAAUUU	UCAUC-3′
	*Reptilian orthoreovirus*[1]*	5′-GUUAUUUU	UCAUC-3′
Aquareovirus genus	*Aquareovirus* A [1]*	5′-GUUUUA	UCAUC-3′
	*Aquareovirus* C [1]*	5′-GUUAUU	UCAUC-3′
	*Aquareovirus* G [1]*	5′-GUUUUA	UCAUC-3′

*Source of genome sequence information is given in square brackets.

### The PRV protein profile deduced from whole-genome sequence analysis

In the present study, the major open reading frames (ORFs) in the 10 PRV genomic segments, identified based on the first methionine of the ORF, vary in length from 315 codons in S4 to 1,290 codons in L2. The lengths of the non-coding regions ranged from 7 to 83 nucleotides at the 5′ end and from 44 to 89 at the 3′ end. The putative PRV gene products calculated from the nucleotide sequence data in this study are shown in Table 3. Only the S1 genome segment is bicistronic, encoding the Outer clamp protein and a nonstructural protein, p13, which is not a FAST protein [10]. In this sense, PRV is similar to *Mammalian orthoreovirus* (MRV), which also does not have a FAST protein and is non-fusogenic. However, MRV differs from PRV in gene coding assignments: in MRV, Core RdRp (λ3) is encoded on segment L1; Core shell (λ1) on segment L3; Outer clamp (σ3) is encoded on S4; Outer fiber (σ1) and NS, other (σ1s) are encoded on S1 (Table 3). Probably the biggest difference is the switch in coding assignments of segments S1 and S4 [2,10]. In most other orthoreoviruses, the Outer fiber protein is encoded on the same bi- or tricistronic S genome segment as the FAST protein and/or a poorly conserved nonstructural protein of unclear function [30].

**Table 3:** Piscine reovirus (PRV) genome coding assignments and protein characteristics

Genome segment*	Molecular size (bp)**	5′UTR (bp)**	3′UTR (bp)**	Protein name***	**Protein size (aa) &mass (kDa)**^**1**^	**pI value**	**Predicted function**
**L1**	3911	18	44	Core shell (T = 1) [λ1]	1282, 141.41 kDa	5.47	major inner capsid protein, Helicase, RNA triphosphatase
**L2**	3935	18	44	Core turret [λ2]	1290, 143.75 kDa	4.81	core spike, guanylyl transferase
**L3**	3916	7	48	Core RdRp [λ3]	1286, 144.24 kDa	8.68	minor inner capsid protein, RNA polymerase
**M1**	2383	21	79	Core NTPase [μ2]	760, 86.09 kDa	8.23	minor inner capsid protein, nucleoside triphosphate phosphohydrolase
**M2**	2179	26	89	Outer shell (T = 13) [μ1]	687, 74.26 kDa	6.27	outer capsid protein, membrane penetration, apoptosis
**M3**	2403	83	61	NS factory [μNS]	752, 83.53 kDa	5.00	NS, genome packaging?
**S1**	1081	28	60	Outer clamp [σ3]	330, 37.08 kDa	7.43	major outer capsid protein, dsRNA binding protein, translation control, modulation of cellular interferon, zinc-binding
				NS, p13 [σ1s]	124, 12.99 kDa	4.88	NS, block cell-cycle progression, cytolytic in PRV
**S2**	1329	21	45	Core clamp [σ2]	420, 45.93 kDa	9.02	major inner capsid protein, morphogenesis?
**S3**	1143	28	50	NS RNA [σNS]	354, 39.07 kDa	7.76	NS, genome packaging?
**S4**	1040	38	54	Outer fiber [σ1]	315, 34.60 kDa	6.04	outer capsid protein (virus attachment), cell tropism, pathways of viral spread *in-vivo*, virulence

*Genome segment nomenclature used by Palacio *et al.*[2] for PRV.

**Values obtained from cDNA sequenced [2; this paper PRV strain 358]. The 3′UTR does not include the stop codon.

***Protein nomenclature used by Key *et al.*[10] for PRV. Homolog of *Mammalian reovirus* protein is given in square brackets.

^1^PRV gene products are calculated from sequence data [2; this paper PRV strain 358].

### Whole-genome sequence comparison to other members of family *Reoviridae*

**Figure 2:**
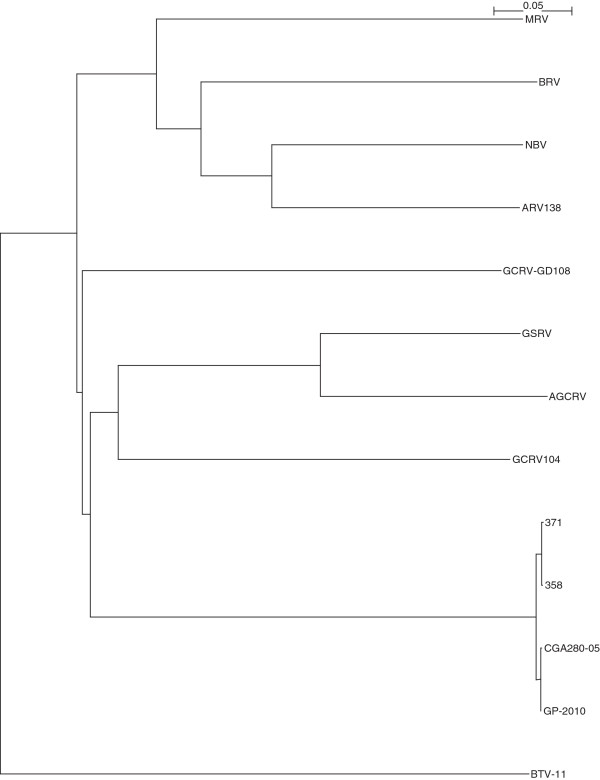
**Phylogeny of homologous segment L1 shared by piscine reovirus (PRV) and selected members of family*Reoviridae*.**

**Figure 3:**
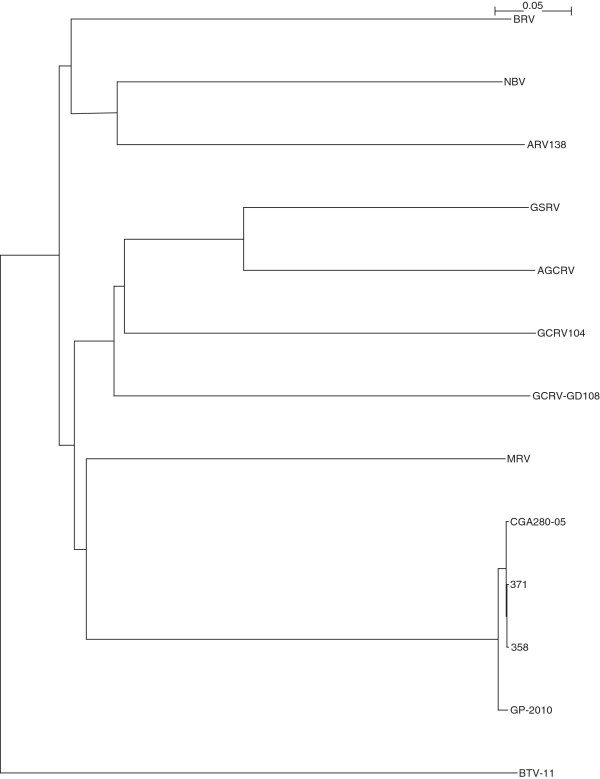
**Phylogeny of homologous segment L2 shared by piscine reovirus (PRV) and selected members of family*Reoviridae*.**

**Figure 4:**
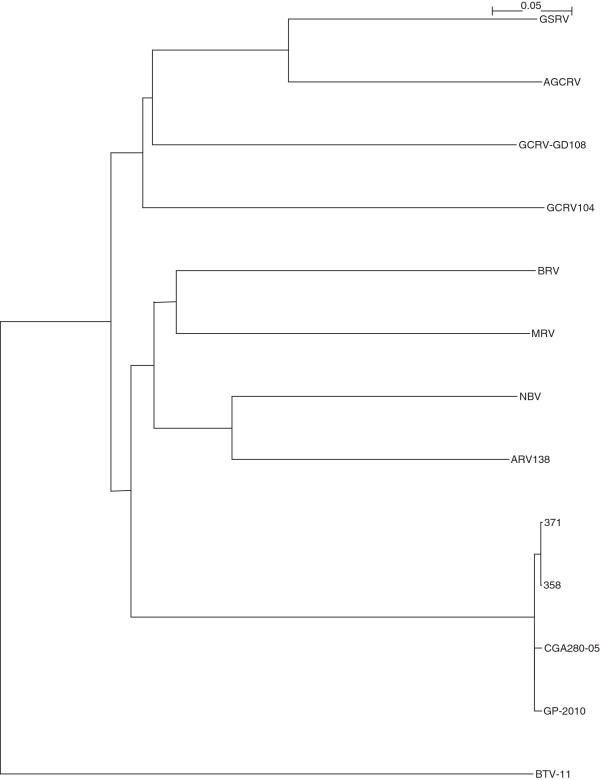
**Phylogeny of homologous segment L3 shared by piscine reovirus (PRV) and selected members of family*Reoviridae*.**

**Figure 5:**
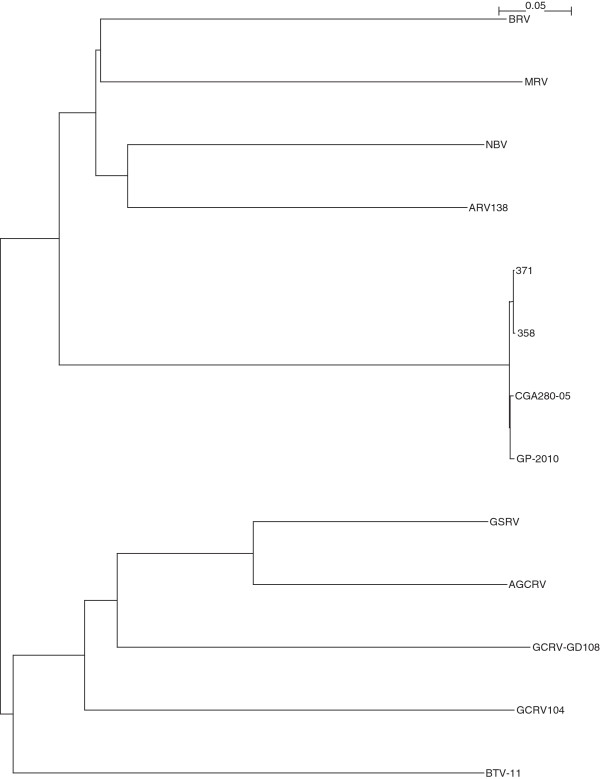
**Phylogeny of homologous segment M1 shared by piscine reovirus (PRV) and selected members of family*Reoviridae*.**

**Figure 6:**
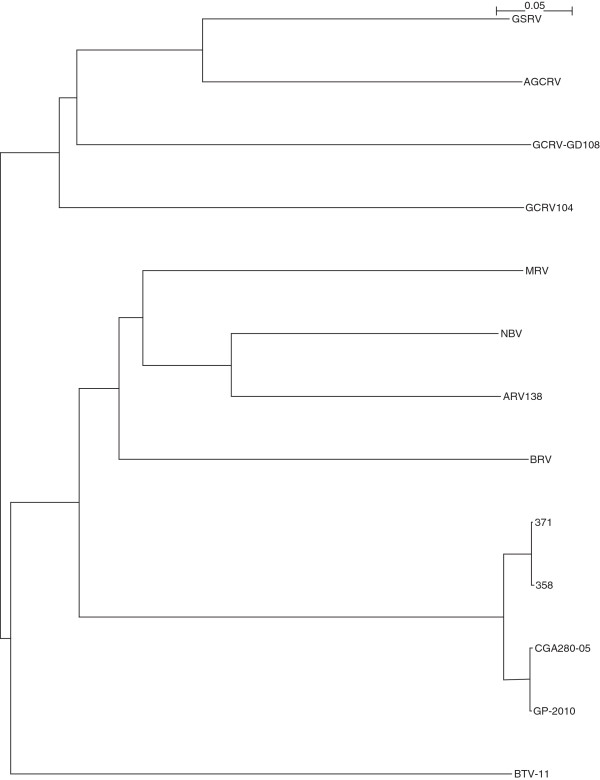
**Phylogeny of homologous segment M2 shared by piscine reovirus (PRV) and selected members of family*Reoviridae*.**

**Figure 7:**
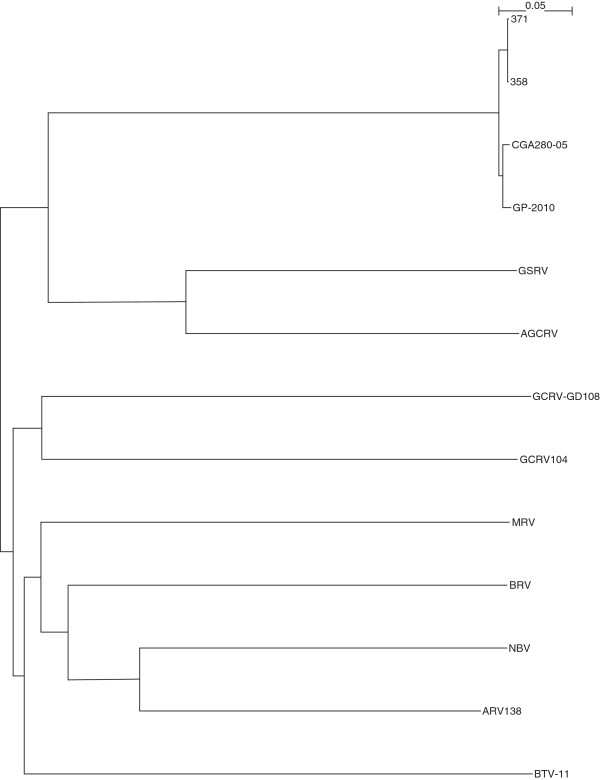
**Phylogeny of homologous segment M3 shared by piscine reovirus (PRV) and selected members of family*Reoviridae*.**

**Figure 8:**
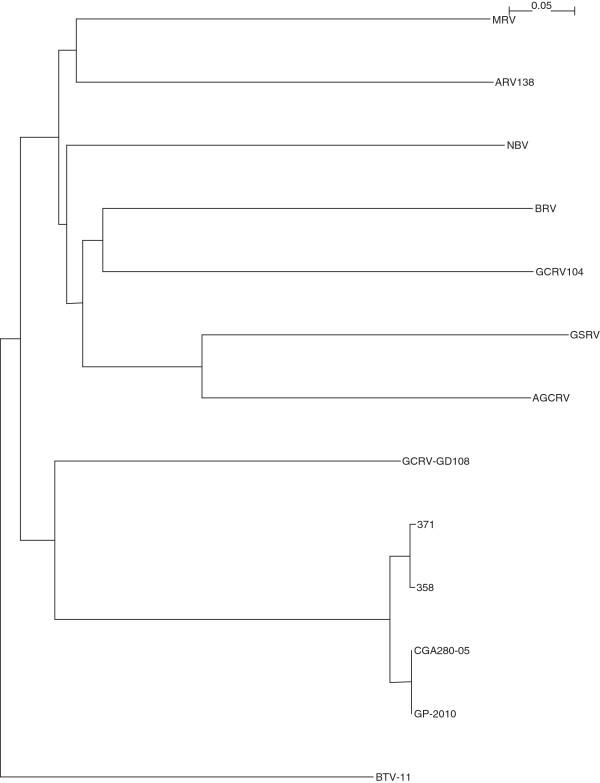
**Phylogeny of homologous segment S1 shared by piscine reovirus (PRV) and selected members of family*Reoviridae*.**

**Figure 9:**
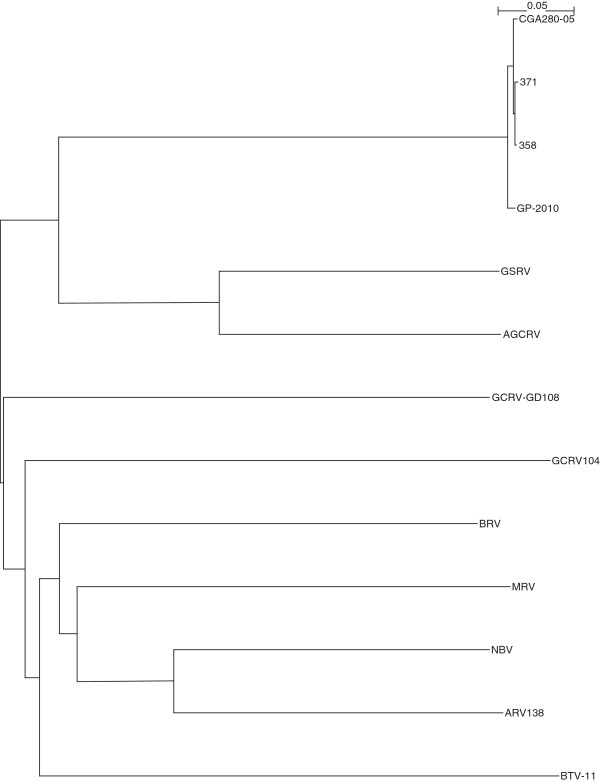
**Phylogeny of homologous segment S2 shared by piscine reovirus (PRV) and selected members of family*Reoviridae*.**

**Figure 10:**
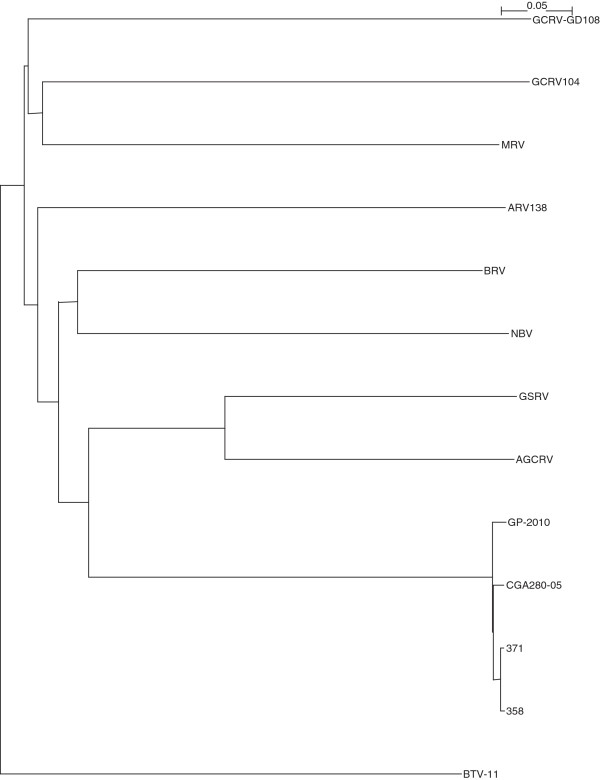
**Phylogeny of homologous segment S3 shared by piscine reovirus (PRV) and selected members of family*Reoviridae*.**

The complete sequencing of Canadian PRV isolates 358, 371, and the Chilean PRV isolate CGA280-5 in the present study enabled us to elaborate the taxonomic grouping of the PRV isolates, and the phylogenetic relationships between *Orthoreovirus*, *Aquareovirus*, and PRV at the genome level. Nucleotide sequences of 13 selected members of family *Reoviridae*, belonging to *Orthoreovirus*, *Aquareovirus*, PRV, and the Bluetongue virus as the outgroup sequence used in the analyses are shown in (Additional file 3: Table S3). Because the equivalent PRV segment S4 gene (Outer fiber protein) is not present in *Aquareovirus* genus, we restricted our analysis to segments homologous to PRV L1, L2, L3, M1, M2, M3, S1, S2, and S3, using a segment to segment comparison approach as done by Palacios *et al.*[2]. For the 13 isolates (Additional file 3: Table S3), we generated a phylogenetic tree for each of the 9 segments L1, L2, L3, M1, M2, M3, S1, S2, and S3. These trees are shown in Figures 2, 3, 4, 5, 6, 7, 8, 9 and 10. All the 9 trees show that PRV isolates cluster in a separate group. In 3 of the 9 trees (i.e., segments homologous to PRV L3, M1, and M2), isolates belonging to *Orthoreovirus*, *Aquareovirus*, and PRV grouped in clearly separate clusters, and would therefore clearly delineate PRV as a new genus separate from both *Orthoreovirus* and *Aquareovirus*. In 3 out of the 9 trees (i.e., segments homologous to PRV L1, M3, and S2), all the isolates inside the *Orthoreovirus* genus are in a separate group. In one tree (i.e., segment homologous to PRV L2), only the isolates inside the *Aquareovirus* genus are in a separate group. In 2 out of the 9 trees (i.e., segments homologous to PRV S1 and S3), none of the three groups of isolates (*Orthoreovirus*, *Aquareovirus*, and PRV) clearly clustered in a separate group although the PRV isolates formed a tight cluster. Thus while the distinction between *Orthoreovirus* genus and *Aquareovirus* genus is not consistent, the distinction between PRV isolates and these two genera is very consistent. These observations further argue for assigning PRV to a new genus within the family *Reoviridae*.

While use of concatenated sequences for phylogenetic analyses [10] for a segmented dsRNA virus may be of limited value because of issues of reassortment and how these may influence phylogenetic groupings, it was also attempted in this study as such a phylogenetic tree is more objective and comprehensively reflects the relationships among the different isolates in *Reoviridae*. Because the lengths of these concatemers were in the range of 18kbp to 23kbp, it was difficult to make sure they aligned well across the whole length. To improve the alignment quality, we first created an alignment for every homologous segment of the 13 isolates (Additional file 3: Table S3) separately, and then merged all the aligned nine segments together for each of the isolates. In this way, we were able to ensure that all alignments involved sequences of the homologous segments. The alignments were visually checked and a phylogenetic tree generated based on this concatemer alignment is shown in (Additional file 4: Figure S1a). The concatemer alignment was then examined for highly conserved regions, which would strongly support the assumption that the 13 isolates had a common ancestor and justify the current phylogenetic analysis. These highly conserved regions were found in segments homologous to PRV segments L1, L2, and L3. The computer software JALVIEW [31] was then used to extract these regions, which were then used to generate another phylogenetic tree (Additional file 4: Figure S1b). A comparison of Figures S1a and S1b revealed that while slight differences exist, both trees individually and in combination support three major groups: *Aquareovirus* genus, *Orthoreovirus* genus, and PRV isolates, i.e., they also support the classification of PRV as a member of a new genus within the family *Reoviridae*.

The distances inside the tree in Additional file 4: Figure S1a provide more insight about this proposal. The average distance between the isolates (MRV, BRV, NBV, ARV138) in the genus *Orthoreovirus* is 0.526. The average distance between an isolate of genus *Orthoreovirus* and an isolate of genus *Aquareovirus* is 0.619. The average distance between a PRV isolate and an isolate of *genus* Orthoreovirus is 0.588, which is much closer to 0.619 than to 0.526, allowing us to unambiguously conclude that PRV represents a new genus within the family *Reoviridae*.

### Phylogenetic analysis and sequence diversity of PRV genomic segment S1

**Figure 11:**
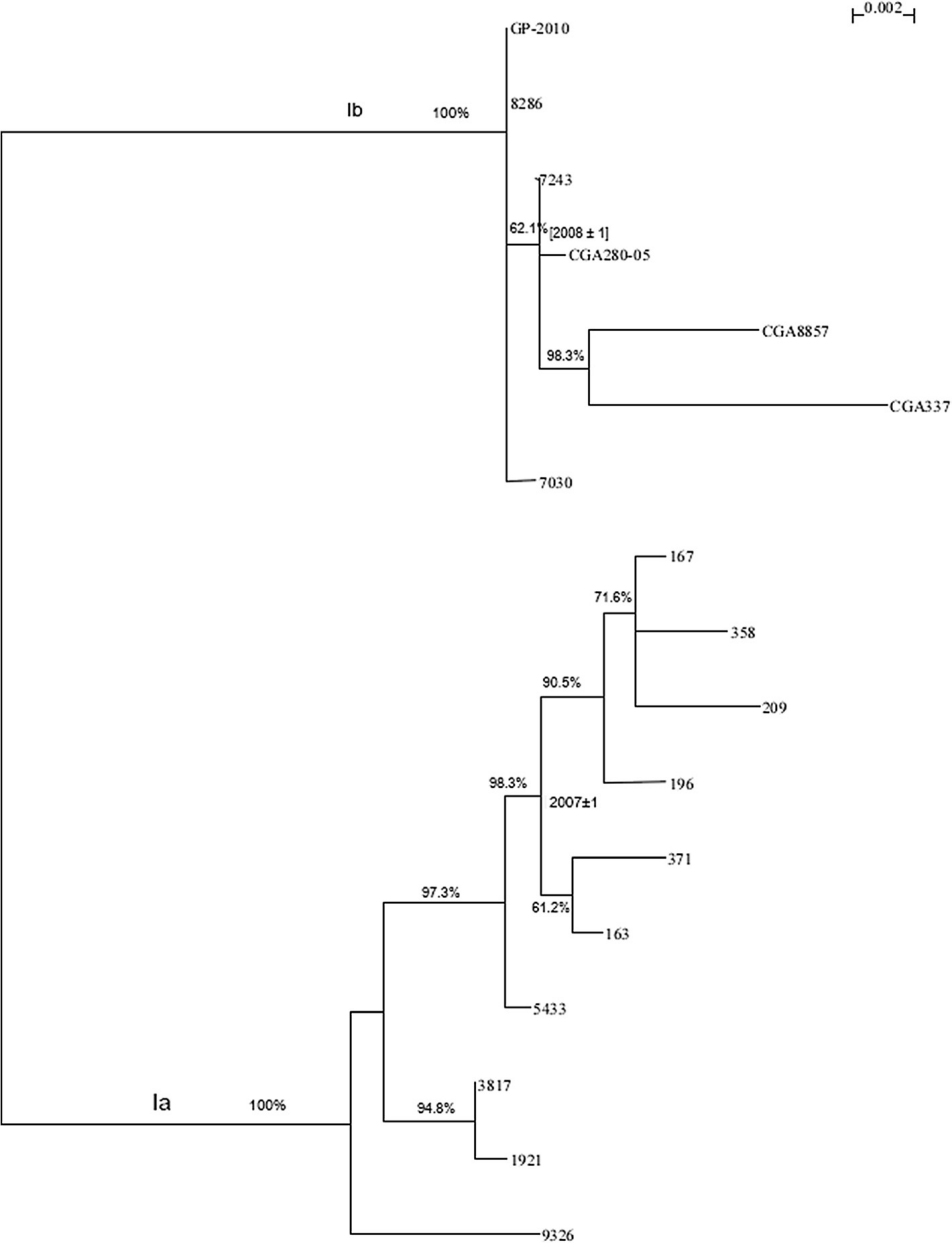
Phylogenetic trees showing the relationships between the different piscine reovirus (PRV) isolates; RNA segment S1 showing the relationships between all PRV isolates.

To determine the genetic relationship between the PRV strains from western Canada, Chile, and Norway, we compared the segment S1 sequences using phylogenetic analyses and percent nucleotide similarities. The sequences of seven of the Norwegian isolates were deposited in the GenBank database as S4 sequences [28] but correspond to S1 sequences of the PRV type strain, Norwegian isolate Reovirus sp. Salmo/GP-2011/NOR [2], and the PRV isolates in the present study. Figure 11 shows the phylogenetic tree generated with these sequences. It shows 4 Norwegian isolates 5433, 3817, 1921, 9326, are very close to the Canadian strains and another 4 Norwegian isolates 7243, 7030, GP-2010, 8286 are very close to Chilean strains. This indicates one PRV genotype in Norway, Genotype I, with two major sub-genotypes, which we designate Ia and Ib, with Canadian PRV strains in sub-genotype Ia and Chilean PRV strains in sub-genotype Ib, both with strong bootstrap support. The Canadian PRV strains form two subgroups, (167, 196, 358, 209, bootstrapping support 90.5%, and 163, 371, bootstrapping support 61.2%). The two PRV sub-genotypes are separated by a relatively long branch (Figure 11), suggesting that they have been evolving independently in Norway. Interestingly, the phylogenetic trees of the individual genome segment nucleotide sequences of *Reoviridae* (Figures 2, 4, 5, 6, 7 and 8, and 10) except for segments homologous to PRV L2 and S2 (Figures 3 and 9, respectively) also seem to support the existence of the two sub-genotypes of PRV.

**Figure 12:**
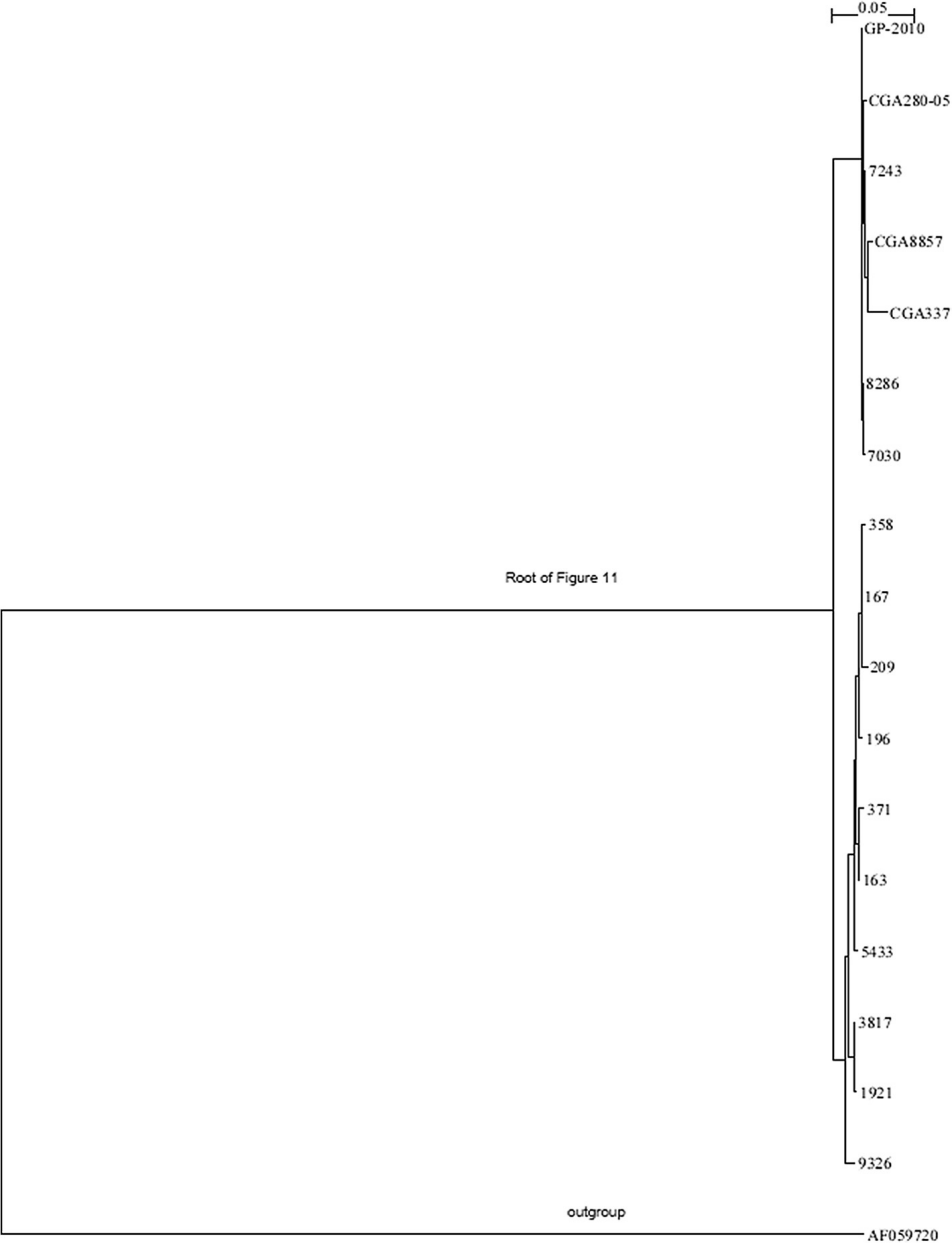
Phylogenetic trees showing the relationships between the different piscine reovirus (PRV) isolates; RNA segment S1 showing the relationships between all PRV isolates with outgroup sequence S3 of Avian reovirus strain 176 (AF059720).

Piscine reovirus segment S1 is bicistronic (Table 3), encoding the Outer clamp protein and a 124-aa protein, designated p13, that induces cytotoxicity [10]. Thus, S1 may be relevant for virulence of PRV. Our sequence analysis of p13 protein showed the Chilean PRV strains had 100% amino acid sequence identity with the Norwegian strain Reovirus sp. Salmo/GP-2010/NOR, whereas the Canadian strains had ≤92.7% amino acid sequence identity with this PRV strain (Additional file 2: Table S2). In the S1 sequence phylogenetic trees (Figures 11 and 12), the Canadian PRV strains are most similar to the Norwegian PRV strains found in Atlantic salmon with HSMI outbreaks from the Lofoten Archipelago of Norway [28]; in contrast, the Chilean PRV strains are most similar to the strains found in Atlantic salmon farms without HSMI outbreaks near Trondheim, Norway [28] (Dr. Torstein Tengs, personal communication). These findings suggest the existence of PRV provides the potential for a HSMI outbreak, but other factors (including environment, stress, PRV/host contact types, PRV infection titre) determine whether a HSMI outbreak actually occurs. This requires further investigation.

Table 4 shows the percent sequence identities on segment S1 between the new Canadian and Chilean PRV strains and the Norwegian PRV isolates reported in the GenBank database. This analysis confirms the phylogenetic analysis (Figures 11 and 12) showing that all Canadian isolates belong to Norwegian sub-genotype Ia and Chilean isolates belong to Norwegian sub-genotype Ib. The Canadian and Norwegian PRV isolates of sub-genotype Ia showed nucleotide sequence identities ≥ 98.1% and amino acid sequence identities ≥ 98.2%. As noted in Table 4, the Chilean PRV S1 sequences 8857 and 337 have inserts relative to the other strains, which contributes to lower sequence identities with other strains. If these two Chilean isolates are excluded, then the Norwegian and Chilean PRV strains of sub-genotype Ib showed nucleotide sequence identities ≥ 98.1% and amino acid sequence identities ≥ 99.4%. The nucleotide and amino acid sequence identities between sub-genotypes Ia and I b strains were ≤ 96.9%. and ≤ 97.0%, respectively, (Table 4).

**Table 4:** **Pairwise sequence comparison of Segment S1 of Canadian, Norwegian and Chilean PRV strains showing two sub-genoypes**^**1**^

**PRV isolate**	**163**	**167**	**196**	**209**	**358**	**371**	5433*	3817*	1921*	9326*	**GP-2010**	8286*	7030*	7243*	**280-5**	**8857**	**337**
163	-	**99.4**	**99.4**	**99.2**	**99.3**	**99.6**	**99.6**	**99.0**	**98.9**	**98.6**	96.8	96.6	96.5	96.5	96.6	94.9	94.8
167	**99.4**	**-**	**99.6**	**99.5**	**99.6**	**99.3**	**99.4**	**98.8**	**98.7**	**98.4**	96.6	96.4	96.3	96.3	96.4	94.7	94.6
196	**99.4**	**99.4**	**-**	**99.4**	**99.4**	**99.3**	**99.4**	**98.8**	**98.7**	**98.4**	96.6	96.4	96.3	96.3	96.4	94.7	94.6
209	**98.8**	**98.8**	**98.8**	**-**	**99.4**	**99.0**	**99.1**	**98.5**	**98.4**	**98.1**	96.3	96.1	96.0	96.0	96.1	94.4	94.3
358	**99.4**	**99.4**	**99.4**	**98.8**	**-**	**99.1**	**99.2**	**98.6**	**98.5**	**98.2**	96.4	96.2	96.1	96.1	96.2	94.4	94.8
371	**98.8**	**98.8**	**98.8**	**98.2**	**98.8**	**-**	**99.4**	**98.8**	**98.7**	**98.4**	96.6	96.4	96.3	96.3	96.4	94.7	94.6
5433*	**99.7**	**99.7**	**99.7**	**99.1**	**99.7**	**99.1**	**-**	**99.2**	**99.1**	**98.8**	96.9	96.9	96.8	96.8	96.7	95.2	95.1
3817*	**99.7**	**99.7**	**99.7**	**99.1**	**99.7**	**99.1**	**100.0**	**-**	**99.9**	**99.0**	96.9	96.9	96.9	96.9	96.8	95.3	95.2
1921*	**99.4**	**99.4**	**99.4**	**98.8**	**99.4**	**98.8**	**99.7**	**99.7**	**-**	**98.9**	96.9	96.9	96.8	96.8	96.7	95.2	95.1
9326*	**99.7**	**99.7**	**99.7**	**99.1**	**99.7**	**99.1**	**100.0**	**100.0**	**99.7**	**-**	96.8	96.8	96.7	96.7	96.6	95.1	95.0
GP-2010	96.7	96.7	96.7	96.1	96.7	96.1	97.0	97.0	96.7	97.0	-	100.0	99.9	99.9	99.8	98.2	98.3
8286*	96.7	96.7	96.7	96.1	96.7	96.1	97.0	97.0	96.7	97.0	100.0	-	99.9	99.9	99.8	98.2	98.3
7030*	96.4	96.4	96.4	95.8	96.4	95.8	96.7	96.7	96.4	96.7	99.7	99.7	-	99.8	99.7	98.1	98.2
7243*	96.7	96.7	96.7	96.1	96.7	96.1	97.0	97.0	96.7	97.0	100.0	100.0	99.7	-	99.9	98.3	98.4
280-5	96.4	96.4	96.4	95.8	96.4	95.8	96.7	96.7	96.4	96.7	99.7	99.7	99.4	99.7	-	98.2	98.3
8857	82.5	82.2	82.2	93.9	82.5	82.2	82.5	82.5	82.2	82.5	86.2	86.2	85.9	86.2	86.2	-	97.4
337	93.0	93.0	93.0	92.3	93.0	92.6	93.3	93.3	93.0	93.3	96.7	96.7	96.3	96.7	96.7	86.6	-

^1^Values above the diagonal are nucleotide sequence identities (%); values below the diagonal are deduced amino acid sequence identities of Outer clamp (σ3) protein (%). Bold text denotes sequence identities among sub-genotype Ia PRV strains.

*Denotes Norwegian PRV “isolates” from Atlantic salmon farms at different points in the life cycle from pre-smolts to fish ready for slaughter [29], available in GenBank Accession Numbers JN991006 (isolate 5433), JN991012 (isolate 3817), JN991007 (isolate 1921), JN991008 (isolate 9326), JN991011 (isolate 8286), JN991010 (isolate 7030), and JN991009 (isolate 7243). PRV isolate Salmo/GP-2010/NOR is the Norwegian PRV sequenced on all 10 genomic segments; its segment S1 sequence is GenBank Accession Number GU994022.

The Chilean PRV sequences 8857 and 337 have inserts relative to the other strains, contributing to the lower sequence identities with other strains.

### Divergence time estimation between Canadian and Chilean PRV and the Norwegian strains

Our analysis using BEAST simulation [32] shows the time when Canadian PRV isolates diverged from Norwegian PRV isolates was between 2006 and 2011; the time when Chilean PRV isolates diverged from Norwegian PRV isolates was between 2003 and 2010. These estimations were based on isolates, collection times (Additional file 1: Tables S1a and S1b) and all the information inside the phylogenetic tree (Figure 11). We also used the program BACKTRACK [33], which reads a phylogenetic tree with evolutionary distances and years of isolation for all the sequences and then generates a time interval for each inner node, to estimate the divergence times. With BACKTRACK, the Canadian isolates diverged from Norwegian isolates between 2007 and 2011; the Chilean isolates diverged from Norwegian isolates between 2004 and 2011. Both algorithms produced a wide range in the estimations. We tried to make the estimations more specific by using the knowledge we have about these sequences. For example, we believe the multiple insertion events of CGA8857 and CGA337 could be caused by some kind of environmental changes and the mutation rates during that period could be significantly higher than normal. Thus, we believe the most likely time when Canadian isolates diverged from Norwegian isolates was between 2006 and 2008, i.e., around 2007 ± 1; the most likely time when Chilean isolates diverged from Norwegian isolates was between 2007 and 2009, around 2008 ± 1. This evolutionary direction was confirmed with several outgroup sequences. The timeline for Canadian PRV is supported by observations that: 1) Heart lesions and HSMI type lesions were reported in British Columbia farmed Atlantic salmon beginning in 2008, in fish that had entered seawater in 2007. The pattern of inflammation in the heart was consistent with systemic immune stimulation; differentials include a bacterial or viral infection [34], and 2) a survey of juvenile Pink salmon “*Oncorhynchus gorbuscha*” in the Broughton Archipelago region of western Canada in April and May 2008 (200 samples in 44 pools) found no PRV when tested with a RT-qPCR assay targeting PRV L1 gene in 2010 [35]. It is not known how the virus could have been transmitted from Norway to Canada since there have never been any authorized direct imports of Atlantic salmon eggs from Norway since 1985; recent imports have been from Washington State-USA (2001) and Iceland (2004–2009) [36]. There is no information about the PRV situation in Washington State or Iceland. Horizontal spread and/or introduction of virus through wild fish migration are not reasonable routes of transmission. The distribution of PRV in Canada is uncertain since there is no national surveillance for it. In Chile, the presence of PRV in farmed Atlantic salmon reared in Chilean seawater was first detected in 2010 and published in a laboratory report in 2011 [26], which cited a high prevalence among the sites located in different areas of the Los Lagos region growing-up macro-zone. PRV could have been introduced to Chile through importation of Atlantic salmon eggs similarly to ISAV [37-39] albeit more recently than ISAV: most Atlantic salmon egg imports to Chile in 2008 were from Norway, and from 2009–2013 have been from Iceland [40]. In Chile, HSMI will be recommended to be included on the List 3 of high-risk diseases [25], thus ensuring active surveillance for it in Chilean aquaculture. To better understand the molecular epidemiology of PRV, it will be necessary to know the situation of PRV in other salmon producing countries and in countries with wild salmonids. Not all countries have surveillance for HSMI; therefore, it may be advisable to consider PRV-HSMI as an emerging disease and initiate its surveillance.

## Conclusions

The present work constitutes the first report of genomic analysis of PRV strains detected in tissue samples obtained from fish in western Canada, and in Chile, extending the geographical range of the characterized virus to Pacific shorelines of both North and South America. Our work suggests PRV entered both Chile and western Canada recently. We provide strong support for classification of PRV as a member of a new genus within the family *Reoviridae*. Our work groups PRV into one genotype, Genotype I, with two major sub-genotypes designated Ia and Ib, with Canadian PRV strains in sub-genotype Ia and Chilean PRV strains in sub-genotype Ib. Taken together, these findings raise awareness on PRV existence outside of Norway so that the aquaculture industry and wild fisheries managers worldwide can become proactive and curtail its international spread, as well as implement mitigation measures regionally and locally.

## Methods

### Fish samples and processing

All samples used in this study were submitted to the laboratory for testing for PRV and other aquatic animal viruses. Samples were either taken from fresh fish and put into microcentrifuge tubes containing RNAlater® (Ambion Inc., Foster City CA) preservative or were immediately placed in sterile whirlpak bags and shipped overnight by courier cold on ice to the testing laboratory; samples, which consisted of individual gill, heart, kidney or pooled gill and heart or kidney, heart and liver tissues, were either bagged individually or pooled (2–3 tissues per pool) for each fish. The fish tissue sample source is detailed in Additional file 1: Table S1. The samples from western Canada were either harvest samples of marine-farmed Atlantic salmon or wild fish samples from fish caught live under sport, scientific or First Nation licenses in regions where Atlantic salmon aquaculture sites were present (Additional file 1: Table S1a).

Each tissue (or pool of tissues) was weighed and macerated to a 10% suspension w/v in phosphate buffered saline (PBS) with 10x antibiotics. The specimen supernatant was used for RNA extraction. The samples from Chile were from marine-farmed Atlantic salmon after seawater transfer (Additional file 1: Table S1b) and were collected in RNAlater® preservative. Samples preserved in RNAlater® (Ambion Inc) were first washed three times with PBS and then homogenized as described above prior to total RNA extraction.

### RT-PCR and nucleic acid sequencing

Total RNA was isolated using a modified total RNA extraction protocol with the RNeasy® mini Kit (QIAGEN). Briefly, total RNA was isolated from samples using 1.25 ml of TRIZOL Reagent (Invitrogen) and 375 μl of sample volume as previously described [41]. The Viral RNA mini Kit (QIAGEN) was also utilized on selected samples following the manufacturer’s recommended protocol. In all cases, the extracted RNA was eluted in 20–50 μl of nuclease-free water, and RNA yields were quantified and purity analysed using the OD260/280 ratio and a NanoDrop ND-1000 spectrophotometer (Thermo Scientific). The eluted RNA was tested immediately following quantitation, or was stored frozen at −80°C until use.

RT-qPCR was run on the LightCycler 480 (Roche Applied Science), version 4.0. The crossing point (Cp) or threshold cycle (Ct) was determined by use of the maximum-second-derivative function on the LightCycler software release 1.5.0. The OneStep RT-PCR kit (QIAGEN) was employed for all RT-qPCR reactions according to the manufacturer’s specifications.

Sample RNA quality was based on RT-qPCR for elongation factor 1 alpha (ELF-1α) as internal control targeting either Atlantic salmon ELF-1α (GenBank accession number BT072490) or Chinook salmon ELF-1α (GenBank accession number FJ890356) carried out using Roche LightCycler® 480 RNA master Hydrolysis Probe kit (Roche Diagnostics). The following primers and probes were used.

For Atlantic salmon EF1α:

ASELF1α Forward – 5′- CGT GAC ATG AGG CAG ACA GT-3′;

ASELF1α Reverse – 5′- CGG CCT TAA CAG CAG ACT TTG-3′;

ASELF1α Probe – 5′-TGC TGT CGG TGT CAT CAA GGC T-3′; and

For Chinook salmon ELF1α:

CSELF1α Forward – 5′- GGT CAC CAC CTA CAT CAA GAA GA-3′;

CSELF1α Reverse – 5′- CCA ACC AGA GAT GGG CAC AAA G-3′;

CSELF1α Probe – 5′-TGG CTA CAA CCC TGC CAC TGT C-3′.

The probes were labelled at the 5′ end with 6-FAM and at the 3′ end with BHQ-1 quencher (Biosearch Technologies Inc.). The final concentrations of primers and probe in each case were 900 nM for each primer and 250 nM for the probe in a final volume of 25 μl. The following thermal cycling parameters were used: 1 cycle of RT for 3 min at 63°C followed by denaturation at 95°C for 3 s, and 45 cycles of denaturation at 95°C for 15 s, annealing and detection at 60°C for 1 min and extension at 72°C for 1 s. Ct values above 40 and no Ct values were defined as negative and these samples would be considered unfit for further testing if after re-extraction and repeated RT-qPCR yielded the same results.

The RT-qPCR assay for PRV used the primer-probe set sequences developed by Haugland *et al*. [42] targeting the PRV L1 gene. The primers were PRV-F– 5′-CCC CAT CCC TCA CAT ATG GAT A-3′and PRV-R– 5′-GGT GAA ATC ATC GCC AAC TCA-3′. The PRV probe, which was labelled at the 5′ end with 6-FAM and at the 3′ end with BHQ-1 quencher (Biosearch Technologies Inc.) was 5′-ATG TCC AGG TAT TTA CC-3′. The reaction conditions were the same as used by Palacios *et al.*[2], but with 8 μl of template RNA. The following concentrations were used: 400 nM primer, 300 nM probe and 1.25 mM MgCl_2_. The following thermal cycling parameters were used: 1 cycle of RT for 30 min at 50°C followed by denaturation at 94°C for 15 min, and 45 cycles of denaturation at 94°C for 15 s, annealing at 54°C for 30 s and amplification and detection at 72°C for 15 s. Samples to be considered positive had Ct values up to 40 and with an exponential curve; Ct values between 40.1 and 45 were considered suspicious, and a sample was negative if there was no Ct value.

Because the laboratory did not have a PRV isolate from cell culture to use as positive control, samples with positive Ct values were further tested in classic RT-PCR targeting the 3′ portion of genome segment L1 with the following PCR primer pairs: PRV-L1 For1 – 5′-CAC TCA CCA ATG ACC CAA ATG C-3′; PRV-L1 Rev1 – 5′-TTG ACA GTC TGG CTA CTT CGG-3′ and/or PRV-L1 For2 – 5′-CTG AAC TGC TAG TTG AGG ATG G-3′; PRV-L1 Rev2 – 5′-GCC AAT CCA AAC AGA TTA GG-3′. These PCR primers and those used to amplify the 10 genomic segments of PRV, listed in (Additional file 5: Table S4) were designed based on the published PRV sequences [2]. RT-PCR for the amplification of each viral genome segment was carried out by using the OneStep RT-PCR kit (QIAGEN). Briefly, the reaction mixture contained 1 μl of total RNA, 4 μl of 5X QIAGEN OneStep RT-PCR buffer, 0.8 μl of dNTPs, 0.5 μM (final concentration) of each primer pair, and 0.8 μl of QIAGEN OneStep RT-PCR enzyme mix in a final volume of 20 μl. Thermal cycling conditions were as follows: an initial cycle of 50°C for 40 min and 95°C for 10 min; 40 cycles of 95°C for 30 s, 54°C for 30 sec, 72°C for 70 s; and a final extension cycle of 72°C for 10 min. Amplified products were analyzed by electrophoresis on 1% agarose gel and purified using High Pure PCR Product Purification Kit (Roche). The PCR products were then either directly sequenced or they were cloned into the pCRII vector using a TOPO TA cloning kit (Invitrogen) in preparation for nucleotide sequencing. Plasmid DNA for sequencing was prepared as described before [43]. DNA sequencing was performed as previously described [44] by ACGT Corporation (Toronto, Ontario, Canada). DNA Sequencing was done either directly on RT-PCR products or on plasmid DNA containing the cloned RT-PCR products obtained from reactions using total RNA from tissue samples.

### Sequencing analysis

Similarity analysis was performed using BLAST programs available via the National Center for Biotechnology Information [45] and the FASTA program package for personal computers [46]. Analysis to identify putative ORFs and their predicted amino acid sequences and other protein characteristics was conducted using the Sequence Manipulation suite, version 2 [47].

### Phylogenetic analyses

The Canadian and Chilean PRV sequences used in the phylogenetic analyses are described in (Additional file 2: Table S2). All the Norwegian PRV sequences were obtained from GenBank [29]. Sequences were processed using ClustalX 2.1 [48]. The multiple sequence alignment was manually verified and adjusted to reach high quality alignment. The phylogenetic trees were generated when positions with gaps were excluded and corrections for multiple substitutions were used. Bootstrapping was performed for 1,000 times. In most cases, only the bootstrapping supports higher than 70% were noted. For some important branches, those bootstrapping values a little lower than 70% were also noted. To verify the evolution direction, outgroup sequences were used to determine the root of the phylogenetic trees.

### Divergence time estimation in a rooted phylogenetic tree

BEAST v1.7.5 [32] was used to estimate divergence time. To find the most suitable substitution model, we ran jModelTest 0.1.1 [49] against the aligned sequences. The result shows K80 model [50] is the most suitable model. Based on this result, a similar model, HKY85 model [51], was chosen in the BEAST simulation. We believe the mutation rates among lineages could be different, and the uncorrelated relaxed molecular clock was chosen. Five million simulation steps were performed and enough effective sample sizes (ESSs) were generated. We also used program BACKTRACK [33], which reads a phylogenetic tree with evolutionary distances and years of isolation for all the sequences and then generates a time interval for each inner node, to estimate the divergence times.

## Competing interests

The authors declare that they have no competing interests in this scientific work.

## Authors’ contributions

MJTK isolated total RNA from tissue samples, performed the RT-qPCR for ELF-1α and PRV and developed and performed the classic RT-PCR for 3′ portion of segment L1 of PRV, and helped to write the manuscript. TI designed the PRV PCR primers used to amplify transcripts of all the 10 genome segments, performed the classic RT-PCR and cloned all PCR products for sequencing and helped to write the manuscript. YW performed all the phylogenetic analyses and helped to write the manuscript. AM provided the Canadian samples for diagnostic testing and edited the manuscript. MGG coordinated the laboratory testing in Chile and edited the manuscript. FSBK coordinated all viral testing and DNA sequence analysis and helped to write the manuscript. All authors read and approved the final manuscript.

## Supplementary Material

Additional file 1 **Title: Additional results on the piscine reovirus (PRV) positive samples from Canada and Chile.** Description: Two tables showing RT-qPCR and conventional RT-PCR results of fish tissue samples from Canada and Chile tested for PRV. Click here for file

Additional file 2 **List of new piscine reovirus (PRV) nucleotide sequences and their percent identity to Norwegian isolate Salmo/GP-2010/NOR.** Click here for file

Additional file 5 **Title: List of oligonucleotide primers used in amplification of piscine reovirus (PRV) genome segments.** Description: Table listing oligonucleotide primers. Click here for file

Additional file 3 **GenBank Accession numbers of genome segments of selected members of family *Reoviridae* used in phylogenetic comparison of nucleotide sequences of individual genome segments [**[52]**].** Click here for file

Additional file 4 **Title: Phylogenetic trees showing the relationships between isolates in family *Reoviridae* at the genome-level.** Description: (Figure S1a) Concatenated sequences of nine homologous segments (segment L1, L2, L3, M1, M2, M3, S1, S2, S3) shared by piscine reovirus (PRV) and selected members of family *Reoviridae*, were used to generate a phylogenetic tree. (Figure S1b) Phylogeny of highly-conserved regions of concatemers in Figure S1a. Click here for file
