# Supplementary material for: Whole-genome analysis of piscine reovirus (PRV) shows PRV represents a new genus in family Reoviridae and its genome segment S1 sequences group it into two separate sub-genotypes
Source: Virol J. 2013 Jul 11;10:230. doi: 10.1186/1743-422X-10-230 (PMC3711887; doi:10.1186/1743-422X-10-230)
Supplement: Additional file 1 — Title: Additional results on the piscine reovirus (PRV) positive samples from Canada and Chile. Description: Two tables showing RT-qPCR and conventional RT-PCR results of fish tissue samples from Canada and Chile tested for PRV. [file 1743-422X-10-230-S1.doc]

**Supplementary Table 1a.** RT-qPCR and conventional RT-PCR results of fish tissue samples from Canada tested for piscine reovirus (PRV)^1^

| **Sample ID** | **Sampling (yyyy-mm)** | **Fish species & nature of sample** | **Tissue type & state** | **Internal control**  **ELF-1α Ct values** | **PRV**  **Ct values** |
| --- | --- | --- | --- | --- | --- |
| VT02142012-23 | 2012-02 | Atlantic salmon, harvest sample | Kidney, frozen | Not done | 27.31 |
| VT02292012-163 | 2012-02 | Atlantic salmon, harvest sample | Gill, frozen | Not done | 26.41 |
| VT02292012-167 | 2012-02 | Atlantic salmon, harvest sample | Gill, frozen | Not done | 26.50 |
| VT03202012-177 | 2012-02 | Atlantic salmon, harvest sample | Gill, frozen | 26.78 | 30.06 |
| VT03202012-185 | 2012-02 | Atlantic salmon, harvest sample | Gill, frozen | 28.58 | 26.33 |
| VT03202012-196 | 2012-02 | Atlantic salmon, harvest sample | Gill, frozen | 27.51 | 21.03 |
| VT03202012-209 | 2012-02 | Atlantic salmon, harvest sample | Gill, frozen | 27.73 | 27.76 |
| VT05112012-321 | 2012-05 | Atlantic salmon, harvest sample | Gill, frozen | 27.42 | 31.61 |
| VT05252012-333 | 2012-05 | Cutthroat trout,  live caught | Gill, frozen | 23.84 | 34.55 |
| VT06042012-340 | 2012-05 | Cutthroat trout,  live caught | Gill, frozen | 28.06 | 34.75 |
| VT06062012-358 | 2012-06 | Atlantic salmon, harvest sample | Gill, frozen | 28.35 | 28.12 |
| VT06202012-371 | 2012-06 | Atlantic salmon, harvest sample | Gill & Heart, frozen | 27.34 | 33.36 |
| VT08092012-468 | 2012-07 | Chum salmon,  smolt sample | Gill & Heart, frozen | 19.33 | 34.12 |
| VT08092012-480 | 2012-06 | Steelhead,  harvest sample | Gill, frozen | 17.72 | 37.29 |

^1^All samples were tested for ELF-1α and PRV by RT-qPCR; cycle threshold (Ct) values obtained are listed. Samples were then tested using conventional RT-PCR targeting the different PRV genomic segments to obtain PCR products for DNA sequencing.

**Supplementary Table 1b.** RT-qPCR and conventional RT-PCR results of fish tissue samples from Chile tested for piscine reovirus (PRV)^1^

| **Sample ID** | **Sampling (yyyy-mm)** | **Fish species & nature of sample** | **Tissue type & state** | **Internal control**  **ELF-1α Ct values** | **PRV**  **Ct values** |
| --- | --- | --- | --- | --- | --- |
| CGA337 | 2012-03 | Atlantic salmon, months in seawater unknown | Heart & Kidney fresh | 19.2 | 23.0 |
| CGA558 | 2012-06 | Atlantic salmon, months in seawater  unknown | Heart fresh | 20.5 | 27.6 |
| CGA8857 | 2012-09 | Atlantic salmon, months in seawater  unknown | Kidney, Spleen, & Liver fresh | Not done | 27.6 |
| CGA280-5 | 2011-11 | Atlantic salmon, months in seawater  unknown | Kidney, RNALater® | 30.03 | 21.44 |

^1^All samples were tested for ELF-1α and PRV by RT-qPCR; cycle threshold (Ct) values obtained are listed. Samples were then tested using conventional RT-PCR targeting the different PRV genomic segments to obtain PCR products for DNA sequencing.
