# Supplementary material for: Whole-genome analysis of piscine reovirus (PRV) shows PRV represents a new genus in family Reoviridae and its genome segment S1 sequences group it into two separate sub-genotypes
Source: Virol J. 2013 Jul 11;10:230. doi: 10.1186/1743-422X-10-230 (PMC3711887; doi:10.1186/1743-422X-10-230)
Supplement: Additional file 2 — List of new piscine reovirus (PRV) nucleotide sequences and their percent identity to Norwegian isolate Salmo/GP-2010/NOR. [file 1743-422X-10-230-S2.doc]

**Supplementary Table 2.** List of new piscine reovirus (PRV) nucleotide sequences and their percent identity to Norwegian isolate Salmo/GP-2010/NOR

| **Genome segment** | **PRV “isolate”** | **GenBank accession no. [28]** | **Nucleotide identity (%) with PRV isolate Salmo/GP-2010/NOR^1^** | **Amino acid identity (%) with PRV isolate Salmo/GP-2010/NOR^2^** |
| --- | --- | --- | --- | --- |
| **L1** | 23 | KC795577 | 99% (partial 909 bp) | 100% (303 aa) |
|  | 163 | KC795578 | 99% (partial 1045 bp) | 99.7% (342 aa) |
|  | 167 | KC795579 | 99% (partial 917 bp) | 99.7% (305 aa) |
|  | 177 | JX502837 | 99% (partial 937 bp) | 100% (311 aa) |
|  | 185 | JX502838 | 99% (partial 937 bp) | 99.7% (311 aa) |
|  | 196 | KC795580 | 99% (partial 1045 bp) | 98.8% (342 aa) |
|  | 209 | JX502839 | 99% (partial 937 bp) | 99.7% (311 aa) |
|  | 321 | KC795581 | 99% (partial 937 bp) | 100% (311 aa) |
|  | 333 | KC795582 | 99% (partial 937 bp) | 99.7% (311 aa) |
|  | 340 | KC795583 | 99% (partial 937 bp) | 100% (311 aa) |
|  | 358 | KC715679 | 99% (3911 bp) | 99.8% (1282 aa) |
|  | 371 | KC776256 | 99% (3911 bp) | 99.7% (1282 aa) |
|  | 468 | KC795584 | 99% (partial 937 bp) | 99.7% (311 aa) |
|  | 480 | KC795585 | 99% (partial 937 bp) | 98.4% (311 aa) |
|  | CGA280-5 | KC795565 | 99% (3911 bp) | 99.8% (1282 aa) |
| **L2** | 163 | KC795586 | 97% (partial 1009 bp) | 98.8% (330 aa) |
|  | 358 | KC715680 | 98% (3935 bp) | 99.2% (1290 aa) |
|  | 371 | KC776257 | 98% (3935 bp) | 99.3% (1290 aa) |
|  | CGA280-5 | KC795566 | 98% (3934 bp) | 99.5% (1290 aa) |
| **L3** | 23 | KC795587 | 99% (partial 1052 bp) | 99.7% (348 aa) |
|  | 163 | KC795588 | 99% (partial 1052 bp) | 98.9% (348 aa) |
|  | 358 | KC715681 | 99% (3916 bp) | 99.8% (1286 aa) |
|  | 371 | KC776258 | 99% (3916 bp) | 99.5% (1286 aa) |
|  | CGA280-5 | KC795567 | 99% (3916 bp) | 99.5% (1286 aa) |
|  | CGA337 | KC782500 | 99% (partial 397 bp) | 100% (131 aa) |
|  | CGA558 | KC790987 | 97% (partial 394 bp) | 100% (83 aa) |
|  | CGA8857 | KC782502 | 99% (partial 374 bp) | 99.2% (126 aa) |
| **M1** | 23 | KC795589 | 99% (partial 954 bp) | 100% (311 aa) |
|  | 163 | KC795590 | 99% (partial 954 bp) | 99.7% (311 aa) |
|  | 167 | KC795591 | 99% (partial 954 bp) | 99.4% (311 aa) |
|  | 321 | KC795592 | 99% (partial 954 bp) | 99.4% (311 aa) |
|  | 358 | KC715682 | 99% (2383 bp) | 99.6% (760 aa) |
|  | 371 | KC776259 | 99% (2383 bp) | 99.9% (760 aa) |
|  | CGA280-5 | KC795568 | 99% (2383 bp) | 99.6% (760 aa) |
| **M2** | 163 | KC795593 | 97% (partial 1108 bp) | 98.9% (360 aa) |
|  | 167 | KC795594 | 97% (partial 1108 bp) | 99.4% (360 aa) |
|  | 358 | KC715683 | 97% (2179 bp) | 99.4% (687 aa) |
|  | 371 | KC776260 | 97% (2179 bp) | 99.4% (687 aa) |
|  | CGA280-5 | KC795569 | 99% (2179 bp) | 99.4% (687 aa) |
| **M3** | 23 | KC795595 | 98% (partial 796 bp) | 99.6% (237 aa) |
|  | 163 | KC795596 | 98% (partial 796 bp) | 99.2% (237 aa) |
|  | 167 | KC795597 | 98% (partial 796 bp) | 99.6% (238 aa) |
|  | 321 | KC795598 | 98% (partial 796 bp) | 100% (237 aa) |
|  | 358 | KC715684 | 99% (2403 bp) | 99.6% (752 aa) |
|  | 371 | KC776261 | 99% (2403 bp) | 99.6% (752 aa) |
|  | CGA280-5 | KC795570 | 99% (2403 bp) | 99.6% (752 aa) |
| **S1** | 163 | KC473452 | 97% (1081 bp) | 96.7% (330 aa)/92.7% (124 aa) |
|  | 167 | KC795599 | 97% (1081 bp) | 96.7% (330 aa)/91.9% (124 aa) |
|  | 196 | KC795600 | 97% (1081 bp) | 96.7% (330 aa)/91.9% (123 aa) |
|  | 209 | KC795601 | 96% (1081 bp) | 96.1% (330 aa)/91.9% (124 aa) |
|  | 358 | KC473453 | 96% (1081 bp) | 96.7% (330 aa)/91.9% (124 aa) |
|  | 371 | KC473454 | 97% (1081 bp) | 96.1% (330 aa)/92.7% (124 aa) |
|  | CGA280-5 | KC795571 | 99% (1081 bp) | 99.7% (330 aa)/100% (124 aa) |
|  | CGA337 | KC782501 | 98% (partial 1007 bp) | 96.7% (299 aa)/100% (124 aa) |
|  | CGA8857 | KC790988 | 99% (partial 999 bp) | 86.2% (269 aa)/100% (124 aa) |
| **S2** | 163 | KC795602 | 99% (partial 828 bp) | 98.9% (269 aa) |
|  | 167 | KC795603 | 99% (partial 828 bp) | 100% (269 aa) |
|  | 358 | KC715685 | 99% (1329 bp) | 99.5% (420 aa) |
|  | 371 | KC776262 | 99% (1329 bp) | 99.3% (420 aa) |
|  | CGA280-5 | KC795572 | 99% (1329 bp) | 100% (420 aa) |
| **S3** | 163 | KC795575 | 99% (1143 bp) | 99.7% (354 aa) |
|  | 358 | KC715686 | 98% (1143 bp) | 99.2% (354 aa) |
|  | 371 | KC776263 | 99% (1143 bp) | 99.7% (354 aa) |
|  | CGA280-5 | KC795573 | 99% (1143 bp) | 99.7% (354 aa) |
| **S4** | 163 | KC795576 | 99% (1040 bp) | 94.0% (315 aa) |
|  | 358 | KC715687 | 99% (1040 bp) | 94.3% (315 aa) |
|  | 371 | KC776264 | 99% (1040 bp) | 93.7% (315 aa) |
|  | CGA280-5 | KC795574 | 99% (1040 bp) | 93.7% (315 aa) |

^1^Percent nucleotide sequence identity obtained using blastn suite [18] over region sequenced (# nt compared in base pairs shown in brackets).

^2^Percent amino acid sequence identity obtained using FASTA program [30] (# amino acid residues compared shown in brackets). Genome segment S1 has two open reading frames (330 aa for Outer clamp protein and 124 aa for p13), hence the two values for each PRV isolate.
