# Supplementary material for: Whole-genome analysis of piscine reovirus (PRV) shows PRV represents a new genus in family Reoviridae and its genome segment S1 sequences group it into two separate sub-genotypes
Source: Virol J. 2013 Jul 11;10:230. doi: 10.1186/1743-422X-10-230 (PMC3711887; doi:10.1186/1743-422X-10-230)
Supplement: Additional file 5 — Title: List of oligonucleotide primers used in amplification of piscine reovirus (PRV) genome segments. Description: Table listing oligonucleotide primers. [file 1743-422X-10-230-S5.doc]

**Supplementary Table 4.** The oligonucleotide primers used in amplification of piscine reovirus (PRV) genome segments

| **Segment** | **GenBank**  **Accession**  **Number** | **Forward primer** | | **Reverse primer** | | **Target (nt)** |
| --- | --- | --- | --- | --- | --- | --- |
|  |  | **Name** | **Sequence (5'-3')** | **Name** | **Sequence (5'-3')** |  |
| **L1** | GU994013 | PRV-L1-F1 | GATAATGTTTGTTTTGCCA | PRV-L1-R1 | CAGTGAGAACTTGACGCAAC | 1-1045 |
|  |  | PRV-L1-F2 | CTAACCACGCTTTTACCTACTC | PRV-L1-R2 | GTTGCGAGGTTGGATGAAG | 988-2091 |
|  |  | PRV-L1-F3 | CGATGAACAATGCCAGTATG | PRV-L1-R3 | CAATCCTTCAAGCAGTTCAC | 2000-2973 |
|  |  | PRV-L1-F4 | AGTAGCCAGACTCGTCAATG | PRV-L1-R4 | GATGAAGTTGTCATGTTTGTG | 2904-3911 |
| **L2** | GU994014 | PRV-L2-F1 | GATAATTGTAACGACGAAATGG | PRV-L2-R1 | GTATCACCTCACGACGACTTAG | 1-1009 |
|  |  | PRV-L2-F2 | GGAACTGTGTTGGCTAATGG | PRV-L2-R2 | GACAACCTCAACCAAATAGTC | 940-1912 |
|  |  | PRV-L2-F3 | CTACTGGAGGTTCCTTTGTG | PRV-L2-R3 | GATTTCCAGCTTACTCACAAC | 1836-2847 |
|  |  | PRV-L2-F4 | CCACTGGTGTTGTGAGTAAGC | PRV-L2-R4 | GATGAAGAAGGAACGGCCTA | 2819-3936 |
| **L3** | GU994015 | PRV-L3-F1 | GATAATAATGGAGAAACCTAAAGC | PRV-L3-R1 | CTAAGTCAGAAGCATCCTCAATC | 1-1052 |
|  |  | PRV-L3-F2 | CGTTTCAGATTGGATGGTG | PRV-L3-R2 | GTCCCAACATTGATTCCATC | 959-1902 |
|  |  | PRV-L3-F3 | GAAGGTATGGATGGAATCAATG | PRV-L3-R3 | GTAGTACCCAGGCAGAACTCC | 1874-2908 |
|  |  | PRV-L3-F4 | GTCCAATGAATCTACCTTCTTC | PRV-L3-R4 | GATGAAGAAGGATCGGCC | 2830-3916 |
| **M1** | GU994017 | PRV-M1-F1 | GATAATAACTCCTTTGCCACC | PRV-M1-R1 | GGAAACGAGCAGATCACAAC | 1-943 |
|  |  | PRV-M1-F2 | GATTCCTTTTGGACGAGTG | PRV-M1-R2 | AACAGTGCATACGTGTCAAAC | 879-1560 |
|  |  | PRV-M1-F3 | GTATGCCTGTCATCATTCGTG | PRV-M1-R3 | GATGAAAATCTCTTAAGCCC | 1474-2383 |
| **M2** | GU994016 | PRV-M2-F1 | GATAAATTTGTTTAACAGGCTTG | PRV-M2-R1 | CTCTCACCACCTAGATCCATTG | 1-1108 |
|  |  | PRV-M2-F2 | GAACTCTGGCATTGCATCTG | PRV-M2-R2 | GATGAAGATTTCTCGTTCGG | 1041-2179 |
| **M3** | GU994018 | PRV-M3-F1 | GATAAAGCTTACGACACGTGAC | PRV-M3-R1 | GTCAGAACATCACAATCTACACG | 1-796 |
|  |  | PRV-M3-F2 | GCTCCATTCGTGTAGATTGTG | PRV-M3-R2 | CTCACTTCCATGTCAGCAATC | 766-1627 |
|  |  | PRV-M3-F3 | CCCAGAAGGATTCTCTCATTG | PRV-M3-R3 | GATGAGGAGGGGAGCTCAC | 1516-2404 |
| **S1** | GU994022 | PRV-S1-F1 | GATAAAGACTTCTGTACGTGAAAC | PRV-S1-R1 | GATGAATAAGACCTCCTTCC | 1-1081 |
| **S2** | GU994019 | PRV-S2-F1 | GATAAATTTGTTGGTGACGATATG | PRV-S2-R1 | CAAACGAACGAATGTGGC | 1-828 |
|  |  | PRV-S2-F2 | CAATACATCGCACGAACAGAG | PRV-S2-R2 | GATGAAGAGGCGTGCTGAC | 636-1329 |
| **S3** | GU994020 | PRV-S3-F1 | GATAATTTTGATTGCATACATTC | PRV-S3-R1 | GATGAAGAGATGTTCGATTGTATG | 1-1143 |
| **S4** | GU994021 | PRV-S4-F1 | GATAAAGATCTTAACCGCAGC | PRV-S4-R1 | GATGAAAAACAGGCTTACCG | 1-1040 |
