# Supplementary material for: Whole-genome analysis of piscine reovirus (PRV) shows PRV represents a new genus in family Reoviridae and its genome segment S1 sequences group it into two separate sub-genotypes
Source: Virol J. 2013 Jul 11;10:230. doi: 10.1186/1743-422X-10-230 (PMC3711887; doi:10.1186/1743-422X-10-230)
Supplement: Additional file 3 — GenBank Accession numbers of genome segments of selected members of family Reoviridae used in phylogenetic comparison of nucleotide sequences of individual genome segments [[52]]. [file 1743-422X-10-230-S3.doc]

**Supplementary Table 3.** GenBank Accession numbers of genome segments of selected members of family *Reoviridae* used in phylogenetic comparison of nucleotide sequences of individual genome segments

| PRV  Genome  Segment* | PRV isolates | | | | *Orthoreovirus* genus | | | | *Aquareovirus* genus | | | | Outgroup |
| --- | --- | --- | --- | --- | --- | --- | --- | --- | --- | --- | --- | --- | --- |
|  | Salmo/GP-2010/NOR | 358 | 371 | 280-5 | ARV 138 | NBV | MRV T1L | BRV | GCRV  104 | GCRV  GD108 | AqRVG  AGCRV | AqRVC  GSRV | BTV-11  DE |
| L1 | GU994013 | KC715679 | KC776256 | KC795565 | EU707933 | JF342674 | AF129820 | NC015879 | JN967631 | HQ231200 | NC010586 | NC005168 | JQ972859 |
| L2 | GU994014 | KC715680 | KC776257 | KC795566 | EU707937 | JF342672 | AF378003 | NC015877 | JN967629 | HQ231198 | NC010584 | NC005166 | JQ972854 |
| L3 | GU994015 | KC715681 | KC776258 | KC795567 | EU707935 | JF342673 | M24734 | NC015878 | JN967630 | HQ231199 | NC010585 | NC005167 | JQ972851 |
| M1 | GU994017 | KC715682 | KC776259 | KC795568 | AY557188 | JF342675 | AF461682 | NC015880 | JN967634 | HQ231208 | NC010589 | NC005171 | JQ972856 |
| M2 | GU994016 | KC715683 | KC776260 | KC795569 | AY750052 | JF342676 | AF490617 | NC015881 | JN967633 | HQ231202 | NC010588 | NC005170 | JQ972857 |
| M3 | GU994018 | KC715684 | KC776261 | KC795570 | AY557190 | JF342677 | AF174382 | NC015882 | JN967632 | HQ231201 | NC010587 | NC005169 | JQ972855 |
| S1 | GU994022 | KC473453 | KC473454 | KC795571 | AF059725 | AF218360 | M13139 | NC015884 | JN967635 | HQ231203 | NC010593 | NC005175 | JQ972852 |
| S2 | GU994019 | KC715685 | KC776262 | KC795572 | AF059717 | AF059718 | M17598 | NC015883 | JN967637 | HQ231205 | NC010591 | NC005173 | JQ972853 |
| S3 | GU994020 | KC715686 | KC776263 | KC795573 | AF059721 | AF059726 | M18389 | NC015886 | JN967636 | HQ231204 | NC010592 | NC005174 | JQ972858 |
| S4 | GU994021 | KC715687 | KC776264 | KC795574 | AF218359 | AF059722 | M14779 | AF406787 | absent | absent | absent | absent |  |

*The order of genome segments for *Orthoreovirus* and *Aquareovirus* [52], and Bluetongue virus 11 (BTV-11, outgroup sequences) correspond to the order of PRV genome segments L1 to S4 to facilitate the order for concatemers (order may vary depending on virus species). ARV = Avian reovirus; NBV = Nelson Bay virus; MRV = Mammalian reovirus; BRV = Baboon orthoreovirus; GCRV104 = Reovirus GCRV104 (new grass carp reovirus strain-GCRV104); GCRV-GD108 = Grass carp reovirus strain GCRV-GD108; AqRVG AGCRV = Aquareovirus G American grass carp reovirus strain AGCRV_PB01-155; AqRVC GSRV = Aquareovirus C Golden shiner reovirus strain; BTV-11 DE = Bluetongue virus type 11 strain DE.
