# Supplementary figures and images for: Whole-genome analysis of piscine reovirus (PRV) shows PRV represents a new genus in family Reoviridae and its genome segment S1 sequences group it into two separate sub-genotypes
Source: Virol J. 2013 Jul 11;10:230. doi: 10.1186/1743-422X-10-230 (PMC3711887; doi:10.1186/1743-422X-10-230)

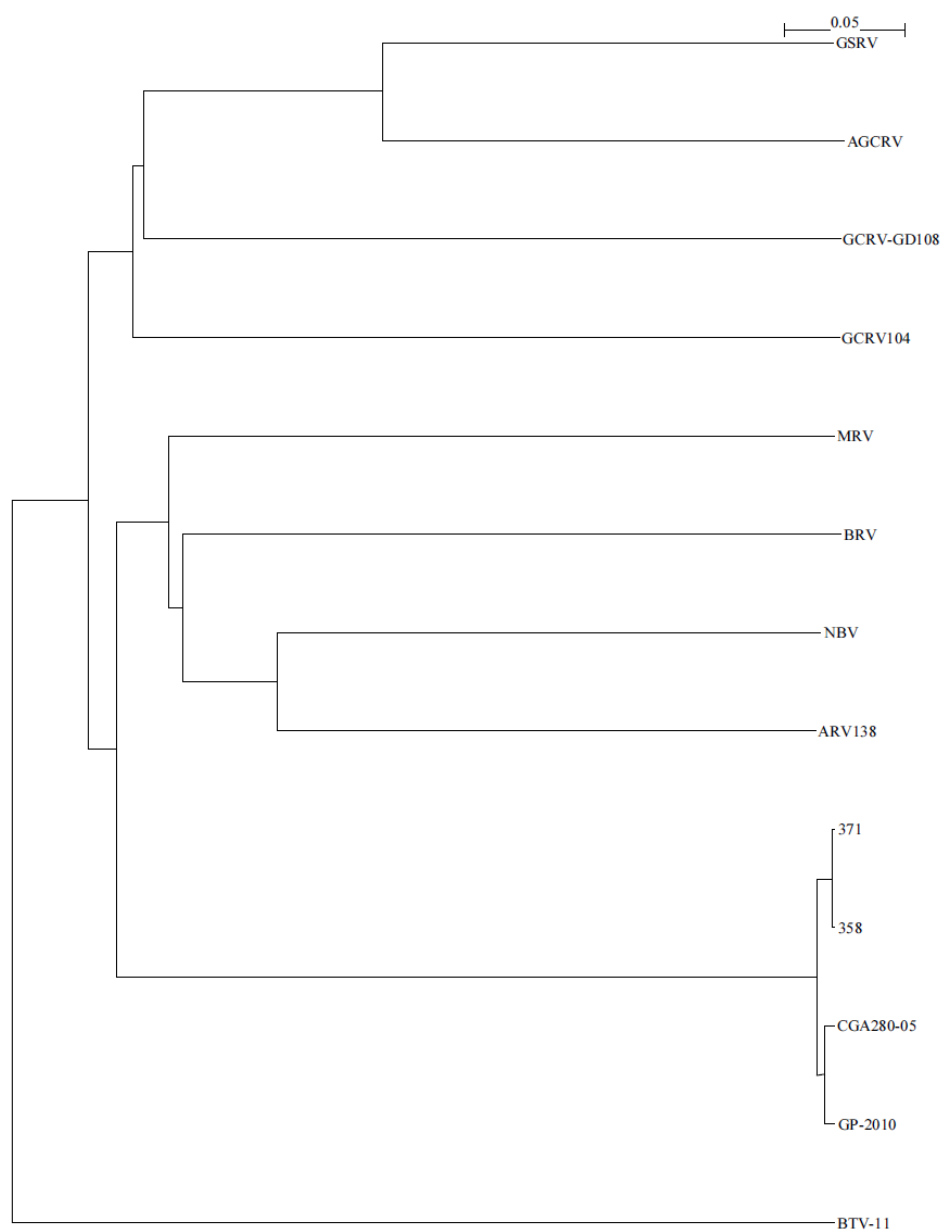

**A**

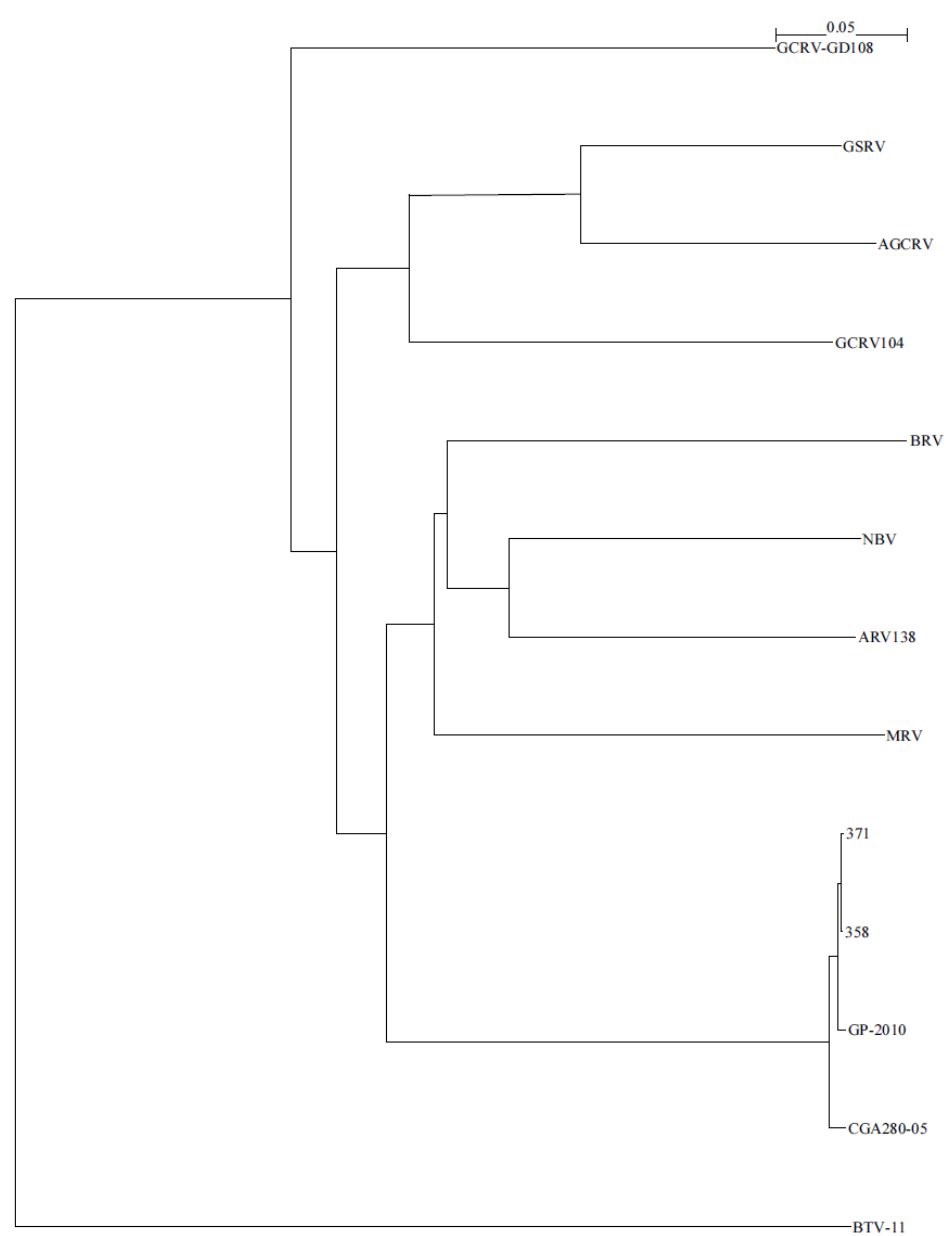

**B**

Supplement: Additional file 4 — Title: Phylogenetic trees showing the relationships between isolates in family Reoviridae at the genome-level. Description: (Figure S1a) Concatenated sequences of nine homologous segments (segment L1, L2, L3, M1, M2, M3, S1, S2, S3) shared by piscine reovirus (PRV) and selected members of family Reoviridae, were used to generate a phylogenetic tree. (Figure S1b) Phylogeny of highly-conserved regions of concatemers in Figure S1a. [file 1743-422X-10-230-S4.pdf]
